# Reductis Ad Absurdum

**Published:** 1895-01

**Authors:** 


REDUCTIO AD ABSURDUM.—Dr. Mark H. Millican has issued a pamphlet, *forninst* castration of criminals. He reviews the several papers which have recently advocated this measure *as a substitute for capital punishment*, and to *stop* the *breed*, and his chief, if not sole argument is,—that to castrate a man destroys his dignity! He says: "It is almost inconceivable to me that enlightened men will destroy a man's dignity." Hanging, we suppose, does not; incarceration in the penitentiary preserves it, and the "dignity" of a masturbator should not, by any means, be interfered with, even though the practice destroy his mind and body. Dignity! Where is the "dignity" of a rapist, an assassin or a murderer? As Dr. Daniel said in his paper ("Castration of Sexual Perverts"): "It is a remarkable civilization that will break a criminal's neck, yet will respect his testicles." And again, "the sexual organs seem to be regarded as something sacred, and the right to exercise them, *ad libitum*—even to produce pauper and criminal offspring—is held as something inalienable;—notwithstanding the offender may have forfeited his citizenship, and with it, all other rights, civil, religious and political."

Dr. Millican reduces the subject to an absurdity; he inferentially deprecates a *race* of "undignifieds"; fears that the *offspring* (?) of the castrated will—Weismann to the contrary notwithstanding—be born without those elements of "dignity," and *theirs*, in turn, will hand down the acquired state,—without a moment's reflection as to how races are propagated, and that to prevent the propagation of criminals is one of the objects sought. Go and poultice your head, doctor. A race of eunuchs!—Oh—that is rich!

---

**Denver** now has a bacteriological laboratory. Dr. La Garde is in charge, and will make a research in all cases of infectious disease occurring in Denver. All cities should follow the example. Such laboratories are a necessity.

